# Vascular Aging and Arterial Stiffness

**DOI:** 10.5935/abc.20170091

**Published:** 2017-09

**Authors:** Luana de Rezende Mikael, Anelise Machado Gomes de Paiva, Marco Mota Gomes, Ana Luiza Lima Sousa, Paulo César Brandão Veiga Jardim, Priscila Valverde de Oliveira Vitorino, Maicon Borges Euzébio, Wátila de Moura Sousa, Weimar Kunz Sebba Barroso

**Affiliations:** 1 Liga de Hipertensão Arterial - Faculdade de Medicina - UFG, Goiânia, GO - Brazil; 2 Hospital do Coração, Maceió, AL - Brazil; 3 Pontifícia Universidade Católica de Goiás, Goiânia, GO - Brazil

**Keywords:** Hypertension, Blood Pressure, Pulse Wave Analysis, Vascular Stiffness, Vascular Remodeling

## Abstract

Cardiovascular diseases (CVD) account annually for almost one third of all deaths worldwide. Among the CVD, systemic arterial hypertension (SAH) is related to more than half of those outcomes. Type 2 diabetes mellitus is an independent risk factor for SAH because it causes functional and structural damage to the arterial wall, leading to stiffness. Several studies have related oxidative stress, production of free radicals, and neuroendocrine and genetic changes to the physiopathogenesis of vascular aging. Indirect ways to analyze that aging process have been widely studied, pulse wave velocity (PWV) being considered gold standard to assess arterial stiffness, because there is large epidemiological evidence of its predictive value for cardiovascular events, and it requires little technical knowledge to be performed. A pulse wave is generated during each cardiac contraction and travels along the arterial bed until finding peripheral resistance or any bifurcation point, determining the appearance of a reflected wave. In young individuals, arteries tend to be more elastic, therefore, the reflected wave occurs later in the cardiac cycle, reaching the heart during diastole. In older individuals, however, the reflected wave occurs earlier, reaching the heart during systole. Because PWV is an important biomarker of vascular damage, highly valuable in determining the patient’s global cardiovascular risk, we chose to review the articles on vascular aging in the context of cardiovascular risk factors and the tools available to the early identification of that damage.

## Physiopathogenesis of vascular aging

Currently 17 million deaths per year are estimated to occur due to cardiovascular diseases (CVD), representing one third of all deaths worldwide. Of those CVD, 9.4 million are related to arterial hypertension (AH),^1^ a highly relevant risk factor for stroke, coronary artery disease, heart failure and occlusive peripheral arterial disease.^2^

Arterial hypertension is often associated with other cardiovascular risk factors (CVRF), such as smoking, obesity, high cholesterol levels and type 2 diabetes mellitus (DM), and that association, mainly with DM, significantly increases the risk for micro- and macrovascular complications, as well as the incidence of CVD.^1,3^

Several studies have shown that DM is an independent and important risk factor for functional and structural damage to the arterial wall, resulting in early arterial stiffness.^4,5^ The combination of those CVRF, mainly AH and DM, contributes to potentiate vascular damage and early arterial aging.^6^

**Figure 1:**
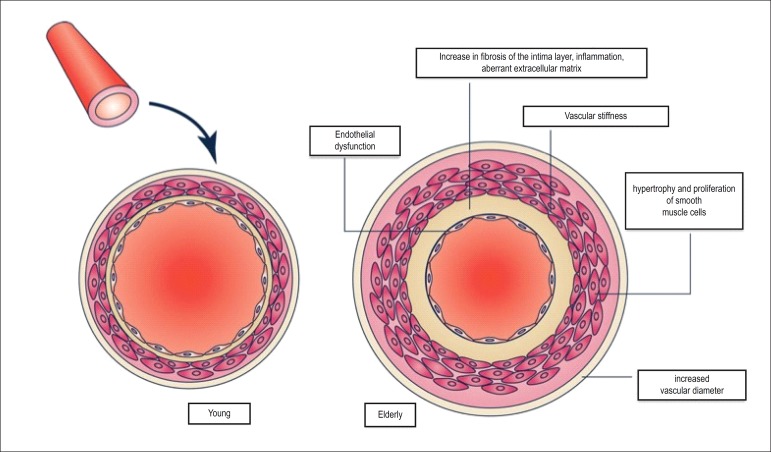
Pathophysiology of vascular aging.^9^

Some theories explain the normal aging process, and, can be generally divided into evolution and physiological or structural theories. From the cardiovascular viewpoint, the major theories include oxidative stress, production of free radicals, neuroendocrine changes and genetic predisposition. The confluence of those factors, acting mainly on myocytes and arterial media-intima layer, increases ventricular and vascular stiffness, a phenomenon closely related to the cardiovascular aging process^7^ (Figure 1).

In the arterial bed, the major structural and functional changes result from calcification, wall diameter increase and elasticity loss, leading to collagen deposition and elastin fragmentation in the media layer. That phenomenon is more evident in large arteries, but also occurs in the peripheral vascular bed.^8,9^ All such changes contribute to reduce arterial compliance and its capacity to resist stress.^10^

The physiopathogenesis of that process is related to changes in the mechanical stretching of the arterial wall and its structural changes.^11^ In addition, there is evidence on the association of inflammatory markers and biomarkers with proatherogenic phenomena, which participate in the pathogenesis of vascular damage. Increased levels of C-reactive protein (CRP), an inflammation marker, are present in hypertensive individuals and contribute to target-organ lesions. In addition, adiponectin, a plasma protein derived from adipocytes that is reduced in hypertensive individuals and related to the glucose metabolism, acts as an antiatherogenic endogenous factor, its reduction being associated with increased cardiovascular risk. Other inflammatory markers and biomarkers described are nuclear factor-kappa B (NF-KB) and insulin growth factor-1 (IGF-1).^9,12^

In addition, age-related changes are associated with the generation of oxygen-reactive species, inflammation, endothelial dysfunction, and calcium and phosphate metabolism disorders.^10^ Those changes depend on genetic characteristics, and vary in different populations. They reflect differences in nutritional characteristics, physical activity, smoking habit, cholesterol and glucose blood levels, and other risk factors known to affect arterial stiffness.^13,14^

Inflammatory mediators participate actively in mechanisms of vascular damage and atherosclerotic disease, their levels being increased in all AH stages. That association accelerates the vascular aging process. In addition, increased CRP levels can reduce the levels of endogenous nitric oxide, an important vasodilator related to the functional regulation of the compliance of large arteries *in vivo*.^12,15^ Thus, the inflammatory and proatherogenic activation, mediated by several biomarkers in the presence of classical CVRF, contributes to worsen CV outcomes.^9,12,16^

The association of genetic, metabolic and inflammatory characteristics with cardiovascular risk phenotypes has been increasingly studied, and some genes that catalyze the process of early vascular aging have been identified.^9^

Finally, the aging phenomenon comprises changes related to a decrease in arterial elasticity and consequent increase in both arterial stiffness and systolic blood pressure (BP) levels. From the physiopathological viewpoint, the decrease in elastin amount, and the increase in collagen amount and in arterial intima-media thickness precede the endothelial damage, and can indirectly identify vascular damage at an initial phase.^7,9,17^

## Arterial stiffness as a consequence of vascular aging

At each heartbeat, a pulse wave is generated and travels along the arterial bed until finding peripheral resistance or any bifurcation point, generating a new reflected wave back to the heart. The velocity of that reflected wave and the phase of the cardiac cycle in which it happens (systole or diastole) depend on peripheral vascular resistance, elasticity, mainly of large arteries, and central BP, being related to major cardiovascular outcomes.^16,17^

In young individuals, arteries are more elastic. Thus, the reflected wave is slow and reaches the heart during diastole, increasing the diastolic pressure and improving coronary perfusion.^18^ In addition, the reflection of the wave returns part of the pulsatile energy to the central aorta, where it is dissipated, limiting the transmission of the pulsatile energy to the periphery and preventing damage to microcirculation.^19^ With vascular aging, pulse wave velocity (PWV) increases, resulting in an early reflection of that wave, which reaches the heart during systole. This increases systolic BP, with a consequent increase in cardiac workload and a reduction in coronary perfusion.^18,19^

The arterial stiffness role in the development of CVD has been more emphatically studied in the past years, its use being recommended in guidelines to improve cardiovascular risk stratification.^20-22^

## Assessment of vascular aging

Vascular aging can be assessed by use of arterial stiffness analysis. Several invasive and non-invasive methods have been described for that purpose. The most widely used and validated techniques involve PWV assessment.^23^

**Figure 2:**
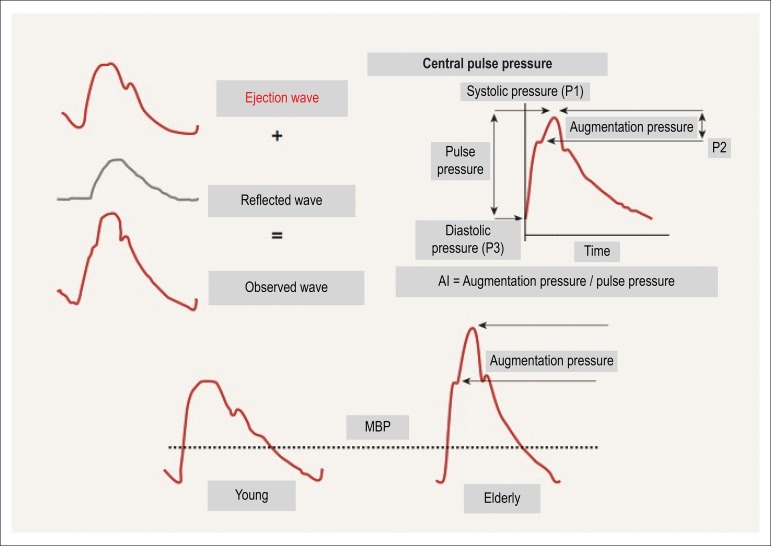
Blood pressure curve with description of its major components. AI: Augmentation Index; MBP: Mean Blood Pressure.^28^

The PWV measurement is considered gold standard to evaluate arterial stiffness. Other methods, such as measuring central systolic BP (cSBP) and augmentation index (AI) (Figure 2), are under greater influence of pathophysiological conditions, medications, heart rate and age, which make them less reliable.^16,23^

Carotid-femoral PWV is a clinically relevant measure of velocity along the aortoiliac trajectory, because the aorta and its first branches are closely related to the left ventricle, and correlate with most of the physiopathogenic effects of arterial stiffness.^16,24^

The carotid-femoral PWV analysis is gold standard for arterial stiffness assessment, because there is large epidemiological evidence of its predictive value for cardiovascular events, and it requires little technical knowledge to be performed. In addition, PWV can be measured in a point. The method for that has been validated, and consists in calculating, by use of transference with calibration, systolic BP/diastolic BP (SBP/DBP) with mean BP/DBP (MBP/DBP), being feasible and having better cost-benefit ratio for clinical practice.^16,21,25^

In addition, PWV bears a strong correlation to age and BP, in which the elastic properties of the arterial wall are reduced, with consequent increase in vascular stiffness.^7,26,27^

## Clinical applicability of vascular aging assessment

Assessment of arterial elasticity (compliance) is clinically important as it correlates with the pathogenesis of a large spectrum of cardiovascular and non-cardiovascular outcomes, such as cerebral white matter lesions and several types of cognitive deficits, such as Alzheimer’s disease, and kidney dysfunction.^28-32^

Vascular cognitive impairment (VCI), a term created to comprise a heterogeneous group of cognitive disorders that share a vascular etiology, including both dementia and cognitive impairment without dementia, has gained importance. This might result from its likely increasing prevalence in next decades, due to population aging and increase in life expectancy consequent to better CVD control.^32^ In addition, VCI increases morbidity, disability and health costs for the elderly, reducing their quality of life and life expectancy.^7,33^

As compared to Alzheimer’s disease, VCI is associated with 50% lower mean survival (6-7 years versus 3-4 years), higher health costs and comorbidity rates. Thus, its primary and secondary prevention is highly important, being usually approached as stroke prevention and changes in vascular risk factors, such as AH, dyslipidemia, DM, obesity and sedentary lifestyle. Better knowledge and early identification of the vascular aging process and of related biomarkers can contribute to improve prevention.^33,34^

The biological aging process is always associated with arterial stiffness, which is accelerated by arterial hypertension. The relationship between arterial stiffness and BP is more complex, being currently understood as bidirectional, because an increase in the vascular distension pressure causes an increase in arterial stiffness, and, conversely, an increase in stiffness can lead to SBP elevation. The relationship between arterial stiffness and BP can also be influenced by antihypertensive drugs, which, by reducing BP, can benefit vascular health. Thus, the interpretation of arterial stiffness data has to consider the patients’ clinical characteristics, such as age, prevalence of comorbidities, use of medications, lifestyle and genetic factors.^35^

In addition to the dominant effect of aging, other physiological and pathophysiological conditions are associated with the increase in arterial stiffness and change in the behavior pattern of the reflected pulse wave: physiological conditions (low birth weight, menstrual cycle, menopause); genetic characteristics (family history of hypertension, DM or myocardial infarction, genetic polymorphisms); CVRF (sedentary lifestyle, obesity, smoking habit, AH, dyslipidemia, glucose intolerance, metabolic syndrome, types 1 and 2 DM); and CVD and non-cardiovascular diseases (different stages of kidney failure, rheumatoid arthritis, systemic vasculitis, systemic lupus erythematosus).^16,19^

Regarding DM and AH, the arterial wall undergoes several biomechanical changes that, from the theoretical viewpoint, can increase arterial stiffness.^36^ In addition, adiponectin has been associated with aortic stiffness in patients with DM.^12^ Another study comparing different procedures to measure PWV to evaluate arterial stiffness in patients with DM has concluded that further investigation is required to clarify its usefulness in those patients, reinforcing PWV as the gold-standard method in that population.^37^ A systematic review assessing the relationship of PWV with several CVRF has shown that 52% of the studies found a positive association between increased PWV and DM.^38^

It is worth noting that arterial stiffness data provide direct evidence of damages in target organs, PWV being considered a biomarker of vascular damage,^17^ which is important in determining the patient’s global cardiovascular risk, considering that the classical risk scores, mainly in the intermediate risk stratum, perform badly to predict cardiovascular outcomes.^21,22^ Traditionally used scores are based on well-established risk factors easily obtained; however, although at least one of those traditional risk factors is present in most patients who have a cardiovascular event, they can be found in patients who will not have an early event.^6,39^

Furthermore, CVD are preceded by an asymptomatic phase. Thus, patients with subclinical damages are at higher risk to develop symptomatic disease, reflecting a possible susceptibility to traditional risk factors. The most recent guidelines on AH have recommended the use of biomarkers to improve the accuracy of cardiovascular risk stratification, especially in patients at intermediate risk.^21,22^

Of the major biomarkers, PWV stands out, which, when added to the classical cardiovascular risk stratification, can reclassify individuals to higher strata, implicating in changes in the management aimed at higher cardiovascular protection.^40,41^

Thus, vascular aging analysis in the risk stratification context can improve the assessment and definition of the management of those patients, and can represent a useful strategy to reduce both absolute and residual risks, because it enables the identification of early damage and the proper treatment already in the cardiovascular *continuum* phase.^42^

## References

[1] World Health Organization (2013). A global brief on hypertension: silent killer, global public health
crisis.

[2] Wu Y, Tai ES, Heng D, Tan CE, Low LP, Lee J (2009). Risk factors associated with hypertension awareness, treatment,
and control in a multi-ethnic Asian population. J Hypertens.

[3] Faria AN, Zanella MT, Kohlman O, Ribeiro AB (2002). Treating diabetes and hypertension in the obese
patient. Arq Bras Endocrinol Metab.

[4] van der Meer RW, Diamant M, Westenberg JJ, Doornbos J, Bax JJ, de Roos A (2007). Magnetic resonance assessment of aortic pulse wave velocity,
aortic distensibility, and cardiac function in uncomplicated type 2 diabetes
mellitus. J Cardiovasc Magn Reson.

[5] Naka KK, Papathanassiou K, Bechlioulis A, Kazakos N, Pappas K, Tigas S (2012). Determinants of vascular function in patients with type 2
diabetes. Cardiovasc Diabetol.

[6] Cecelja M, Chowienczyk P (2009). Dissociation of aortic pulse wave velocity with risk factors for
cardiovascular disease other than hypertension: a systematic
review. Hypertension.

[7] Cefalu CA (2011). Theories and mechanisms of aging. Clin Geriatr Med.

[8] Stratton JR, Levy WC, Caldwell JH, Jacobson A, May J, Matsuoka D (2003). Effects of aging on cardiovascular responses to parasympathetic
withdrawal. J Am Coll Cardiol.

[9] Costantino S, Paneni F, Cosentino F (2016). Ageing, metabolism and cardiovascular disease. J Physiol.

[10] Benetos A, Salvi P, Lacolley P (2011). Blood pressure regulation during the aging process the end of the
'hypertension era'?. J Hypertens.

[11] Nigam A, Mitchell GF, Lambert J, Tardif JC (2003). Relation between conduit vessel stiffness (assessed by tonometry)
and endothelial function (assessed by flow-mediated dilatation) in patients
with and without coronary heart disease. Am J Cardiol.

[12] Tsioufis C, Dimitriadis K, Selima M, Thomopoulos C, Mihas C, Skiadas I (2007). Low-grade inflammation and hypoadiponectinaemia have an additive
detrimental effect on aortic stiffness in essential hypertensive
patients. Eur Heart J.

[13] Avolio AP, Deng FQ, Li WQ, Luo YF, Huang ZD, Xing LF (1985). Effects of aging on arterial distensibility in populations with
high and low prevalence of hypertension: comparison between urban and rural
communities in China. Circulation.

[14] Lanzer P, Boehm M, Sorribas V, Thiriet M, Janzen J, Zeller T (2014). Medial vascular calcification revisited: review and
perspectives. Eur Heart J.

[15] Wilkinson IB, Qasem A, McEniery CM, Webb DJ, Avolio AP, Cockcroft JR (2002). Nitric oxide regulates local arterial distensibility in
vivo. Circulation.

[16] Townsend RR, Wilkinson IB, Schiffrin EL, Avolio AP, Chirinos JA, Cockcroft JR, American Heart Association Council on Hypertension (2015). Recommendations for improving and standardizing vascular research
on arterial stiffness: a scientific statement from the American Heart
Association. Hypertension.

[17] Vlachopoulos C, Xaplanteris P, Aboyans V, Brodmann M, Cifkova R, Cosentino F (2015). The role of vascular biomarkers for primary and secondary
prevention. A position paper from the European Society of Cardiology Working
Group on peripheral circulation: Endorsed by the Association for Research
into Arterial Structure and Physiology (ARTERY) Society. Atherosclerosis.

[18] Nichols W, O'Rourke M, Viachopoulos C (2011). McDonald's blood flow in arteries: theoretical, experimental and
clinical principles.

[19] Safar ME, Levy BI, Struijker-Boudier H (2003). Current perspectives on arterial stiffness and pulse pressure in
hypertension and cardiovascular diseases. Circulation.

[20] Laurent S, Cockcroft J, Van Bortel L, Boutouyrie P, Giannattasio C, Hayoz D, European Network for Non-invasive Investigation of Large
Arteries (2006). Expert consensus document on arterial stiffness: methodological
issues and clinical applications. Eur Heart J.

[21] Mancia G, Fagard R, Narkiewicz K, Redon J, Zanchetti A, Bohm M (2013). 2013 ESH/ESC Guidelines for the management of arterial
hypertension: the Task Force for the management of arterial hypertension of
the European Society of Hypertension (ESH) and of the European Society of
Cardiology (ESC). J Hypertens.

[22] Malachias MV, Souza WK, Plavnik FL, Rodrigues CI, Brandão AA, Neves MF, Sociedade Brasileira de Cardiologia (2016). 7ª Diretriz brasileira de hipertensão
arterial. Arq Bras Cardiol.

[23] van Sloten TT, Schram MT, van den Hurk K, Dekker JM, Nijpels G, Henry RM (2014). Local stiffness of the carotid and femoral artery is associated
with incident cardiovascular events and all-cause mortality: the Hoorn
study. J Am Coll Cardiol.

[24] Pannier B, Guerin AP, Marchais SJ, Safar ME, London GM (2005). Stiffness of capacitive and conduit arteries: prognostic
significance for end-stage renal disease patients. Hypertension.

[25] Mattace-Raso F, Hofman A, Verwoert GC, Wittemana JC, Wilkinson I, Cockcroft J, Reference Values for Arterial Stiffness Collaboration (2010). Determinants of pulse wave velocity in healthy people and in the
presence of cardiovascular risk factors: 'establishing normal and reference
values'. Eur Heart J.

[26] Greenwald SE, Carter AC, Berry CL (1990). Effect of age on the in vitro reflection coefficient of the
aortoiliac bifurcation in humans. Circulation.

[27] Rizzoni D, Porteri E, Boari GE, De Ciuceis C, Sleiman I, Muiesan ML (2003). Prognostic significance of small-artery structure in
hypertension. Circulation.

[28] Safar ME (2010). Antihypertensive efficacy and destiffening
strategy. Medicographia.

[29] Liao D, Cooper L, Cai J, Toole J, Bryan N, Burke G (1997). The prevalence and severity of white matter lesions, their
relationship with age, ethnicity, gender, and cardiovascular disease risk
factors: the ARIC Study. Neuroepidemiology.

[30] Mitchell GF (2004). Increased aortic stiffness: an unfavorable cardiorenal
connection. Hypertension.

[31] Safar ME, London GM, Plante GE (2004). Arterial stiffness and kidney function. Hypertension.

[32] Kalaria RN, Akinyemi R, Ihara M (2012). Does vascular pathology contribute to Alzheimer
changes?. J Neurol Sci.

[33] Levine DA, Langa KM (2011). Vascular cognitive impairment: disease mechanisms and therapeutic
implications. Neurotherapeutics.

[34] Zoorob RJ, Kihlberg CJ, Taylor SE (2011). Aging and disease prevention. Clin Geriatr Med.

[35] Kotsis V, Stabouli S, Karafillis I, Nilsson P (2011). Early vascular aging and the role of central blood
pressure. J Hypertens.

[36] Brooks BA, Molyneaux LM, Yue DK (2001). Augmentation of central arterial pressure in Type 2
diabetes. Diabet Med.

[37] Lacy PS, O'Brien DG, Stanley AG, Dewar MM, Swales PP, Williams B (2004). Increased pulse wave velocity is not associated with elevated
augmentation index in patients with diabetes. J Hypertens.

[38] Jerrard-Dunne P, Mahmud A, Feely J (2008). Ambulatory arterial stiffness index, pulse wave velocity and
augmentation index--interchangeable or mutually exclusive
measures?. J Hypertens.

[39] Boutouyrie P, Tropeano AI, Asmar R, Gautier I, Benetos A, Lacolley P (2002). Aortic stiffness is an independent predictor of primary coronary
events in hypertensive patients: a longitudinal study. Hypertension.

[40] Laurent S, Briet M, Boutouyrie P (2012). Arterial stiffness as surrogate end point: needed clinical
trials. Hypertension.

[41] Sehestedt T, Jeppesen J, Hansen TW, Rasmussen S, Wachtell K, Ibsen H (2012). Thresholds for pulse wave velocity, urine albumin creatinine
ratio and left ventricular mass index using SCORE, Framingham and ESH/ESC
risk charts. J Hypertens.

[42] Thomopoulos C, Parati G, Zanchetti A (2014). Effects of blood pressure lowering on outcome incidence in
hypertension: 3. Effects in patients at different levels of cardiovascular
risk--overview and meta-analyses of randomized trials. J Hypertens.

